# Using Cell-Based Strategies to Break the Link between Bronchopulmonary Dysplasia and the Development of Chronic Lung Disease in Later Life

**DOI:** 10.1155/2013/874161

**Published:** 2013-01-14

**Authors:** Megan O'Reilly, Bernard Thébaud

**Affiliations:** ^1^Department of Pediatrics, Women and Children's Health Research Institute, Katz Group Centre for Pharmacy and Health Research, University of Alberta, Edmonton, AB, Canada T6G 2E1; ^2^Sprott Centre for Stem Cell Research, Ottawa Hospital Research Institute, 501 Smyth Road, Ottawa, ON, Canada K1H 8L6; ^3^Division of Neonatology, Department of Pediatrics, Children's Hospital of Eastern Ontario (CHEO) and CHEO Research Institute, 401 Smyth Road, Ottawa, ON, Canada K1H 5B2

## Abstract

Bronchopulmonary dysplasia (BPD) is the chronic lung disease of prematurity that affects very preterm infants. Although advances in perinatal care have changed the course of lung injury and enabled the survival of infants born as early as 23-24 weeks of gestation, BPD still remains a common complication of extreme prematurity, and there is no specific treatment for it. Furthermore, children, adolescents, and adults who were born very preterm and developed BPD have an increased risk of persistent lung dysfunction, including early-onset emphysema. Therefore, it is possible that early-life pulmonary insults, such as extreme prematurity and BPD, may increase the risk of COPD later in life, especially if exposed to secondary challenges such as respiratory infections and/or smoking. Recent advances in our understanding of stem/progenitor cells and their potential to repair damaged organs offer the possibility of cell-based treatments for neonatal and adult lung injuries. This paper summarizes the long-term pulmonary outcomes of preterm birth and BPD and discusses the recent advances of cell-based therapies for lung diseases, with a particular focus on BPD and COPD.

## 1. Introduction

Intrauterine and early postnatal environments have been shown to play an influential role in the development and maturation of the lung [1]. Suboptimal conditions that interfere with normal development may result in altered lung structure and function and increase the risk for disease later in life. Alarmingly, the onset of adult lung disease following inadequate development and maturation is becoming apparent at an early age. Recently, Wong and colleagues [2] showed that survivors of moderate-severe bronchopulmonary dysplasia (BPD) presented with emphysema in early adulthood (17–33 years of age). Understanding how the fetus and developing lung responds to intrauterine alterations and adapts to the postnatal environment can teach us about basic biology and the implications for adult lung diseases [3, 4]. 

## 2. Early Life Origins of BPD

Development of the lung throughout gestation is a vital process required for adequate fetal to neonatal transition at birth. As the fetal lung proceeds through its developmental stages *in utero*, it becomes progressively prepared for exposure to the external environment. Successful transition to *ex utero* life at birth is dependent upon the ability of the lungs to effectively function as an organ of gas exchange. Indeed, organs of the developing fetus and newborn infant are extremely plastic and are particularly vulnerable to intrauterine and early postnatal environments. Therefore, being born preterm with structurally immature and surfactant-deficient lungs usually results in exposure to many environmental factors that can impact later lung development and function. It is likely that the long-term pulmonary outcomes of preterm birth and BPD are a result of complex programming mechanisms influenced by both environmental and epigenetic changes that take place during the period of development [1, 5]. Many fetal and postnatal factors associated with preterm birth modulate the pathogenesis of BPD, including severity or prematurity, oxidative stress from supplemental oxygen therapy, ventilator-induced lung injury, fetal and/or postnatal infection or inflammation, and nutrition [6, 7]; therefore, identification of the injurious factors contributing to the development of BPD is often hampered by its multifactorial etiology. 

## 3. Preterm Birth and BPD

Over the past few decades, advances in perinatal care have improved the outcome for infants born extremely preterm (i.e., between 24 and 28 weeks of gestation), and there is currently a rising trend in the rate of survival. However, with the shift in the limit of viability toward a lower gestational age, the task of protecting the more immature lung from injury becomes increasingly challenging. Severity of prematurity is one of the major risk factors for the development BPD [8]. Due to their immature pulmonary surfactant system, immature alveoli, and underdeveloped surface area for adequate gas exchange, almost all extremely preterm infants require prolonged respiratory support to ensure survival [6]; however, this further increases their risk of developing BPD. Furthermore, infants who were born extremely preterm have a high incidence of rehospitalization during their first year of life (over 40% of prematurely born infants), with the most common cause for re-hospitalization being respiratory infection [9]. It has been demonstrated by many studies that prematurely born infants who develop BPD carry a life-long risk of poor pulmonary health; this is discussed in detail later.

## 4. Short- and Long-Term Pulmonary Outcomes of Preterm Birth and BPD

Recent evidence suggests that BPD has long-term respiratory complications that reach beyond childhood into adulthood. Numerous follow-up studies show an increased risk of impaired lung function in infancy, childhood, adolescence, and early adulthood. Few studies have investigated the long-term pulmonary outcome in adults exceeding their early 20's. This is particularly important because it is only now that large populations of very preterm subjects are reaching middle-age adulthood (i.e., 35–45 years of age) and may be at risk of persistent respiratory morbidity.

### 4.1. Pulmonary Outcomes during Infancy

 Several studies have documented abnormal pulmonary function during infancy (up to approximately 2 years of age) following preterm birth and development of BPD [10–15]. These studies indicate that prematurely born infants (both with and without BPD) experience reduced lung function in the first few months of life. Compared to term-born infants, preterm infants with BPD are more likely to be symptomatic with recurrent wheezing [14] and require re-hospitalization during the first 2 years of life due to acute respiratory distress and respiratory tract illness [13, 15]. Functional tests show significantly decreased airway function in prematurely born infants compared to term born controls, including decreased forced expiratory volume in 0.5 seconds (FEV_0.5_) [12, 14], reduced forced expiratory flow rates (FEF_50%_, FEF_75%_, and FEF_25–75%_) [10, 12, 14], reduced functional residual capacity (FRC) [11, 15], and increased lung residual volume (RV) [14]. Reduced pulmonary diffusing capacity has also been demonstrated in clinically stable infants and toddlers with chronic lung disease of prematurity, suggesting impairment of alveolar development, albeit alveolar volume appeared normal [16]. Furthermore, respiration variables of prematurely born infants have been shown to be significantly altered during the first 2 years of life, indicated by a faster breathing frequency [11, 15], greater tidal volume (*V*_*T*_) [11], increased amount of dead space [11], and greater minute ventilation (*V*_*E*_) [11]. There are also considerable differences in compliance and resistance of the respiratory system, with preterm infants presenting with lower compliance and higher resistance compared to term-born controls [11]. 

### 4.2. Pulmonary Outcomes during Childhood

 There is a wealth of information regarding pulmonary outcomes during childhood following preterm birth, with the majority of follow-up studies assessing respiratory symptoms, pulmonary function, and exercise capacity in school-age children (approximately 7 to 12 years of age) [17]. Indeed, many studies indicate that prematurely born children have an increased risk of respiratory symptoms, including cough [13, 18], wheeze [13, 18], and asthma [18, 19]. Assessment of pulmonary function throughout childhood shows that preterm birth increases the risk of impaired lung function, with studies showing significantly impaired respiratory function variables in prematurely born children compared to term-born controls; these impairments are particularly common in individuals who develop BPD following preterm birth. Poor respiratory function variables include decreased FEV_1_ [19–29], reduced FEF_25%–75%_ [19–24, 26, 27, 29, 30], reduced FVC [19–24, 26, 29], decreased total lung capacity (TLC) [19, 21], and increased RV [13, 19, 21, 22, 28], all of which indicate varying degrees of airflow obstruction, as well as decreased diffusing capacity of the lungs [27, 28]. Furthermore, exercise capacity is compromised in ex-preterm children (both with and without BPD) compared to term-born control subjects. In response to maximal exercise tests, exercise performances of prematurely born children were indicative of reduced aerobic power, as shown by a significantly lower maximal heart rate [13, 25], faster breathing frequency [22, 24], smaller *V*_*T*_ [22], reduced maximal oxygen consumption (*V*O_2max⁡_) [21–23, 25], reduced maximal minute ventilation (*V*_*E*max⁡_) [23], and shorter exercise time [23] and distance [21, 25]; these functional differences are consistent with long-term alterations in cardiopulmonary development. Interestingly, oxygen supplementation during the neonatal period in very low birth weight infants has been identified as an independent risk factor for asthma in childhood [31]. 

 Some of the childhood studies described earlier were conducted in children who were born in the presurfactant and preantenatal steroid era [13, 23, 30]. However, the remaining studies discussed earlier were performed in the postsurfactant and postantenatal steroid era, which indicates that even with the introduction of pre and postnatal interventions to reduce the severity of BPD, poor pulmonary outcomes are still evident in childhood, perhaps as a consequence of earlier interference with normal lung development.

### 4.3. Pulmonary Outcomes during Adolescence and Young Adulthood

Poor pulmonary outcomes observed in prematurely born children throughout childhood remain prevalent into adolescence and young adulthood [17]. In comparison to the numerous extensive studies performed in ex-preterm children, there are few studies that have investigated ex-preterm adults older than 20 years. Recent studies of adolescents and adults have assessed pulmonary outcomes from approximately 14 to 22 years of age [32–35]. Respiratory symptoms still persist to late adolescence and early adulthood, with significantly more ex-preterm subjects reporting the occurrence of cough, wheeze, and asthma compared to term-born controls [32–34]. Prematurely born adolescents also had a significantly greater risk of being hospitalized for respiratory problems [32]. As with pulmonary function outcomes in childhood, FEV_1_, FEF_25%–75%_, and FVC all remained significantly lower in late adolescence and young adulthood following preterm birth compared to control subjects [25, 29, 35, 36]. Additionally, exercise capacity remained lower than that of controls, with subjects exhibiting significantly lower maximal heart rate, faster breathing frequency, reduced *V*O_2max⁡_, reduced *V*_*E*max⁡_, and shorter exercise distance in response to exercise tests [25, 35]. Of particular interest is the recent evidence showing higher exhaled breath condensate 8-isoprostane levels in ex-preterm nonBPD and BPD adolescents, indicative of significantly increased oxidative stress in their airways; this suggests the presence of an ongoing airway disease [36]. More alarmingly, studies are surfacing of impaired alveolar development and emphysema at adult age in former preterm infants with BPD [2, 37]. Data from such follow-up studies suggest that very preterm birth, or associated factors, can permanently affect the small conducting airways and gas exchanging region and may contribute to impaired lung function observed further into adulthood.

It is important to note that the adolescent and young adulthood studies described earlier were undertaken with individuals born during the presurfactant era (birth years of the subjects ranged from 1977 to 1985), with the exception of the studies by Kotecha et al., 2012 [29], and Filippone et al., 2012 [36]. Although similar pulmonary outcomes are still observed in the postsurfactant era during childhood, further longitudinal follow-up studies are required from the present survivors of preterm birth who were treated with surfactant to gain a thorough understanding of the long-term pulmonary outcomes. To date, there are limited functional studies that have been undertaken in adolescent and young adult subjects from the postsurfactant era (i.e., infants born after 1990). It is anticipated that in the forthcoming years, further data will become available, since preterm individuals that were the first to be treated with synthetic surfactant are now entering this age range. 

## 5. Neonatal Lung Injury and the Influence of Aging on Lung Structure and Function 

 It is well known that aging is associated with progressive decline in lung function, a result of many age-induced structural alterations to the lung [38, 39]. Increases in the total alveolar airspace volume with enlargement of the alveolar ducts are common consequences of aging and are thought to arise from increases in the size and frequency of interalveolar pores [40]. A reduction in the supporting parenchymal tissue within the lung can result in collapse of the small conducting airways during normal breathing, especially during expiration, potentially causing gas trapping and hyperinflation, more commonly known as “senile emphysema.” The increase in airspace size resulting from loss of supporting tissue and a decrease in elastic recoil are likely the cause of reported increases in lung compliance with aging [38, 39, 41]. Furthermore, aging is thought to be associated with a decline in pulmonary immune function [40], and increases in the number of immune cells in bronchoalveolar lavage fluid have been reported in the aging human population [42]. This evidence of persistent low-grade inflammation in the respiratory tract is thought to cause oxidant-mediated injury to the lung matrix, resulting in a loss of alveoli and impaired gas exchange [42]. 

The recent reports of increased oxidative stress in the airways of ex-preterm nonBPD and BPD adolescents [36] in conjunction with reports of early-onset emphysema in ex-preterm BPD adults [2, 37] suggest that preterm birth and the subsequent development of BPD may accelerate and/or exacerbate the normal age-induced pulmonary changes. Therefore, it is reasonable to consider that early life pulmonary insults, such as preterm birth *per se* and BPD, may increase the risk of chronic obstructive pulmonary disease (COPD) later in life [43], especially if the lungs of ex-preterm BPD adults are exposed to secondary challenges such as respiratory infections and/or smoking. 

Progress toward decreasing the incidence/severity of BPD over the next few years using currently available techniques and strategies is likely (i.e., optimization of antenatal management combined with surfactant and early noninvasive ventilatory support targeting lower oxygen saturations) [44]. However, currently, there is a lack of treatment for both BPD and the chronic lung disease that consequently ensues later in life. Therefore, a further understanding of the mechanisms involved in lung development, injury, and repair are necessary in order to advance toward preventing lung injury and/or promoting lung development/regeneration in extremely and very prematurely born infants. Exciting discoveries in stem cell biology over recent years may offer new insight into the pathogenesis of BPD and, more importantly, open new therapeutic avenues. Consequently, as we advance in the quest to provide therapeutic stem/progenitor cell-based strategies for the prevention and repair of neonatal lung injury, our focus may need to extend to potential therapies for the ex-preterm BPD adult lung exhibiting early-onset emphysema/COPD. Indeed, BPD and COPD exhibit common therapeutic targets [3].

## 6. Therapeutic Potential of Stem/Progenitor Cells for Lung Repair/Regeneration

Recent animal and human studies suggest that damage or depletion of stem/progenitor cells in the developing lung likely contributes to the pathogenesis of both BPD and COPD (including emphysema), thus highlighting the potential of stem/progenitor cell supplementation for the prevention or repair of lung injury. The use of stem/progenitor cells is being increasingly examined in experimental animal models and provides compelling evidence for the beneficial effects of stem cell therapy approaches for a wide variety of lung diseases [45–47]. Given the perturbations of the resident lung stem/progenitor cells in both BPD and COPD, the ideal therapeutic approach involves replenishing the damaged lung with healthy multipotent stem/progenitor cells that could repopulate, repair, and regenerate the injured lung. Indeed, several recent studies have demonstrated promising outcomes using different types of stem/progenitor cell types in animal models of BPD (Table 1) and COPD (Table 2).

### 6.1. Lung Progenitor/Stem Cells in Health and Disease

Observations from our lab in an oxygen-challenged neonatal rat model of BPD have shown significantly reduced numbers of circulating and resident mesenchymal stem cells (MSCs) in the lung [48]. It has also been shown that the umbilical cord blood (UCB) of preterm infants yields a higher amount of endothelial colony-forming cells (ECFCs; a specific subset of endothelial progenitor cells, EPCs) than that of term-born infants; however, those preterm ECFCs exhibited an increased susceptibility to *in vitro* oxygen exposure [49]. Of particular interest is the reduced number of ECFCs in preterm infants who subsequently developed BPD compared to nonBPD preterm infants [50]. 

Similarly, resident lung stem/progenitor cells in the presence of COPD exhibit comparable perturbed characteristics. A recent study by Liu and Xie [51] found that the number of early outgrowth EPCs (a subset of EPCs that exhibit a spindle-like morphology *in-vitro* culture at 4–7 days and express the hematopoietic lineage cell markers CD34, CD133, and VEGFR-2) [52] isolated from patients with COPD was significantly less than that of control subjects. Furthermore, the EPCs of COPD patients exhibited reduced cluster-forming numbers and impaired cell migratory capacity, suggesting that the capacity of EPCs to repair dysfunctional endothelium is compromised in COPD [51]. These dysfunctional characteristics of COPD EPCs were also confirmed in a study by Kim and colleagues [53], where they observed significantly lower colony-forming units and lower migratory capacity of circulating EPCs isolated from patients with emphysema. These findings from BPD and COPD studies highlight the potential of stem/progenitor cell supplementation for repair/regeneration of the lung. 

### 6.2. Therapeutic Potential of Mesenchymal Stem Cells in BPD and COPD

Of the many different types of stem/progenitor cell therapies that have been used in experimental models, MSCs appear to be the most extensively examined cell type. MSCs possess the ability to differentiate and form various mesenchymal cell types, including bone, cartilage, and adipose cells, and can be sourced from the bone marrow, UCB, umbilical cord Wharton's jelly, the placenta, and adipose tissue. 

#### 6.2.1. MSCs in Preclinical Studies for BPD

Administration of bone marrow-derived MSCs, either intratracheally, intravenously, or intraperitoneally, has ameliorated numerous aspects of neonatal lung injury, as evident by mitigation of lung inflammation, prevention of lung vascular damage and alveolar growth arrest, inhibition of lung fibrosis, and improvement in exercise tolerance [48, 54–58]. 

Low engraftment and differentiation of these MSCs into the injured neonatal lung suggest that the potential mechanisms through which MSCs exert their actions are paracrine mediated. These speculations are supported by *in vitro *and *in vivo* studies demonstrating that administration of conditioned media (CdM) from MSCs has the ability to protect alveolar epithelial and lung microvascular endothelial cells from oxidative stress, prevent oxygen-induced alveolar growth arrest, and stimulate a subset of stem/progenitor cells, known as bronchoalveolar stem cells (BASCs), to aid in lung repair [48, 54, 55, 57, 58]. Furthermore, the therapeutic benefits of MSC-CdM may surpass those of MSCs, with *in-vivo *findings indicating a more profound therapeutic effect of MSC-CdM in preventing/repairing lung injury than that of MSCs [54]. 

Compared to the various available MSC sources, UCB represents a very appealing source of MSCs for therapeutic use in the newborn due to its clinically relevant, easily accessible, ethically viable, and readily available source of stem/progenitor cells. Chang and colleagues [59, 60] demonstrated that MSCs obtained from human UCB prevent hyperoxia-induced alveolar growth arrest and alleviate fibrotic changes in the neonatal rat lung. Interestingly, Chang et al. also show that the route of administration may alter the outcome, with intratracheal transplantation resulting in a more prominent attenuation of hyperoxia-induced lung injury than intraperitoneal transplantation [60]. Furthermore, Chang et al. recently demonstrated the dose-dependent effects of human UCB-derived MSCs in the oxygen-challenged neonatal rat lung [59]. This study indicated that intratracheal delivery of a minimum of 5 × 10^4^ cells is required to exhibit efficient antiinflammatory, antifibrotic, and antioxidative effects following hyperoxia-induced lung injury in neonatal rats [59]. In light of these findings, further studies determining the optimal dose of MSCs for potential clinical benefit in human neonates are anticipated. More recently, data from our own lab has demonstrated the beneficial long-term effects of UCB-derived MSCs in an oxygen-challenged rat model of BPD [58]. Long-term assessment of 6-month-old rats showed no adverse effects of either MSC or MSC-CdM therapy and also demonstrated persistent improvements in adult exercise capacity and lung structure [58]. 

#### 6.2.2. MSCs in Preclinical Studies for COPD

Repair of lung injury in experimental models of COPD/emphysema has also been demonstrated by many studies utilizing MSCs, derived from bone marrow, adipose tissue, and umbilical cord Wharton's jelly [61–70]. Huh and colleagues [61] used a cigarette smoke-induced experimental model of COPD/emphysema and showed that bone marrow-derived MSCs and their CdM were capable of restoring the alveolar structure and increased pulmonary vascularity. Zhen and colleagues [62] also used bone marrow-derived MSCs, but in a papain-induced model of COPD/emphysema, and showed alveolar structure improvements. The beneficial effects of MSCs have also been demonstrated in an elastase-induced experimental model of COPD/emphysema, where Katsha and colleagues [65] also showed preservation of the alveolar structure, reduced inflammation, and upregulation of growth factors. The bleomycin-induced adult lung injury model has also been implemented in many studies that demonstrate the beneficial effects of MSCs, as evident by reduced lung fibrosis and modulation of pulmonary inflammation [66–70]. Similar to the use of MSCs in experimental BPD animal models, the proposed mechanism of action is through a paracrine and immunomodulatory effect rather than cell engraftment [71]. 

Apart from MSCs, other reparative cells of interest include EPCs, amnion epithelial cells (AECs), and perivascular cells (PCs). The therapeutic potential of EPCs in neonatal lung injury has been effectively demonstrated in an oxygen-induced BPD mouse model [72]. Treatment of neonatal mice exposed to hyperoxia with intravenously administered bone marrow-derived angiogenic cells (a population of bone marrow myeloid-like precursor cells) showed restoration of the alveolar structure and vessel density to that of control (room air-exposed) levels [72]. Recently, the therapeutic potential of human AECs has been investigated in a sheep model of neonatal lung injury, induced by LPS administration in fetal sheep [73]. Since human AECs are sourced from placentae, which are normally discarded after birth, they present an easily accessible and ethically viable cell therapy candidate. Administration of AECs to fetal sheep exposed to LPS attenuated inflammation-induced changes in lung function and structure and reduced pulmonary inflammation [73]. Of particular interest is the ability of AECs to significantly increase the expression levels of SP-A and -C. The potential therapeutic effects of human AECs have also been examined in bleomycin-induced adult lung injury models [74, 75]. Administration of AECs to adult mice exposed to bleomycin attenuated lung fibrosis, reduced collagen content, and decreased pulmonary inflammation [74, 75]; partial restoration of lung function was also observed following AEC administration [75]. Recently, umbilical cord-derived PCs have been shown to exhibit similar reparative potential to UCB-derived MSCs in a rat model of neonatal lung injury [58]. Umbilical cord-derived PCs, as a whole cell therapy or growth factor producers (i.e., CdM), rescued oxygen-induced arrested alveolar growth and improved long-term lung function [58]. The low engraftment into the lungs indicates that these cells act via immune modulation, rather than cell engraftment and differentiation. More detailed assessment of the therapeutic potential of these cells in other models of neonatal and adult lung injury will be of interest. 

## 7. Summary

The long-term pulmonary outcomes reported in ex-preterm BPD children, adolescents, and adults highlight the potential link between altered early-life development and environment and chronic lung disease in adulthood. Findings from several exciting studies indicate that a variety of stem/progenitor cells can prevent and/or regenerate neonatal and adult lung injury in various experimental models. In terms of neonatal lung injury, additional studies in different animal models of BPD are necessary to broaden the current knowledge and understanding of the therapeutic potential of stem/progenitor cells. In doing so, further evidence for creating a strong rationale for translating this potential breakthrough into the clinic can be generated. Furthermore, by preventing or repairing neonatal lung injury in prematurely born infants, it is possible that the overall risk of COPD/emphysema development in later life may be reduced in this subset of the population. 

## Figures and Tables

**Table 1:** Studies examining the therapeutic effect of stem/progenitor cells in experimental models of neonatal chronic lung disease.

Experimental model	Therapeutic cell or product	Outcomes	Suggested mechanism	References
	Bone marrow-derived MSCs (i.t.)	Improved survival Improved alveolar structure/prevented alveolar arrest Prevented vascular growth arrest Improved exercise capacity Reduced pulmonary hypertension	Engraftment as AT2 Paracrine mechanisms	[48]
	Bone marrow-derived MSCs or CdM (i.v.)	Improved alveolar structure/prevented alveolar arrest Attenuated inflammation Prevented vascular growth arrest Prevented pulmonary hypertension	Paracrine mechanisms Immunomodulatory effects	[54]
	Bone marrow-derived MSCs or CdM (i.v.)	Increased number of BASCs Improved alveolar structure/prevented alveolar arrest	Stimulation of BASCs Paracrine mechanisms	[55]
Hyperoxia-induced neonatal lung injury	Bone marrow-derived MSCs (i.p.)	Improved survival Improved alveolar structure/prevented alveolar arrest Attenuated inflammation Inhibited lung fibrosis	Engraftment as AT2 Reduction in ECM remodeling and fibrosis gene expression (TGF-*β*1, collagen 1*α*, TIMP-1) Anti-inflammatory effects	[56]
	Bone marrow-derived MSC-CdM (i.v.)	Improved alveolar structure Attenuated myofibroblast infiltration Improved lung function Reversed pulmonary hypertention and RV hypertrophy Attenuated pulmonary arterial remodeling Rescued loss of pulmonary blood vessels	Paracrine mechanisms Cytoprotective effects Activation of BASCs	[57]
	hUCB-derived MSCs (i.t.)	Improved survival and growth restriction Improved alveolar structure Attenuated lung fibrosis, inflammation, and ROS activity	Paracrine anti-inflammatory, antifibrotic and antioxidative effects	[59, 60]
	hUCB-derived MSCs and MSC-CdM (i.t.) Umbilical cord-derived PCs and PC-CdM (i.t.)	Prevented and restored impaired alveolar growth Improved lung function and exercise capacity Prevented impaired lung angiogenesis Prevented pulmonary arterial wall remodeling and RV hypertrophy Persistent benefit on lung architecture and exercise capacity at 6 months No adverse effects on lung structure in treated control animals at 6 months	Paracrine mechanisms	[58]
	BMDACs (i.v.)	Improved alveolar structure Improved vascular growth	Paracrine mechanisms	[72]

LPS-induced (i.a.) neonatal lung injury	hAECs (i.t.; i.v.)	Improved alveolar structure Increased surfactant protein expression Attenuated inflammation	Immunomodulatory effects	[73]

Acronyms: AT2: alveolar epithelial type 2; BASC: bronchioalveolar stem cell; BMDAC: bone marrow-derived angiogenic cell; CdM: conditioned media; ECM: extracellular matrix; hAEC: human amnion epithelial cell; hUCB: human umbilical cord blood; i.a.: intraamniotic; i.p.: intraperitoneal; i.t.: intratracheal; i.v.: intravenous; LPS: lipopolysaccharide; MSC: mesenchymal stem cell; PC: perivascular cell; ROS: reactive oxygen species; RV: right ventricle; TGF-*β*1: transforming growth factor-*β*1; TIMP1: tissue inhibitor of metalloproteinase 1.

**Table 2:** Studies examining the therapeutic effect of stem/progenitor cells in experimental models of adult chronic lung disease.

Experimental model	Therapeutic cell or product	Outcomes	Suggested mechanism	References
Cigarette smoke-induced adult lung injury	Bone marrow-derived MSCs, CdM, and BMCs (i.v.)	Restoration of alveolar structure Increased pulmonary vascularity Alleviation of pulmonary hypertension (by BMCs)	Paracrine mechanisms Recruitment of BMCs by donor cells	[61]

Papain-induced adult lung injury	Bone marrow-derived MSCs (i.v.)	Improved alveolar structure	Engraftment and AT2 differentiation Reduced alveolar epithelial apoptosis	[62]

Elastase-induced (i.t.) adult lung injury	Adipose tissue-derived MSCs (i.v. or cultured on PGA and transplanted after LVRS)	Restored gas exchange Improved exercise tolerance	Growth factor release (HGF, VEGF)	[63, 64]
	Bone marrow-derived MSCs (i.t.)	Preservation of alveolar structure Reduced inflammation Upregulated growth factors	Paracrine mechanisms HGF, EGF, and secretory leukocyte protease inhibitor secretion	[65]
	Lung resident multilineage progenitors Sca1^+^CD45^−^CD31^−^ (i.t.)	Improved survival Attenuated alveolar damage	Immunomodulatory effects Paracrine mechanisms	[76]

Bleomycin-induced adult lung injury (i.t.)	Human ESC-derived cells with AT2 epithelial phenotype (i.t.)	Improved body weight and survival Improved arterial oxygen saturation Decreased collagen deposition	Engraftment and AT1 differentiation Paracrine mechanisms	[77]
	Bone marrow-derived MSCs (i.v.)	Reduced fibrosis and inflammation	IL-1 receptor antagonism Decrease in NO metabolites, proinflammatory, and angiogenic cytokines	[66, 68, 70]
	hUC Wharton's jelly-derived MSCs (i.v.)	Reduced fibrosis	Decreased TGF-*β* and TIMP activity Increased MMP-2 activity	[67]
	Bone marrow-derived HSCs ± KGF overexpression (i.v.)	Reduced fibrosis	KGF-induced endogenous AT2 cell proliferation	[78]

Bleomycin-induced adult lung injury (i.n.)	hAECs (i.p.; i.v.)	Reduced fibrosis and collagen deposition Improved lung function Modulated inflammatory response	Anti-inflammatory effects	[74, 75]

Acronyms: AT1: alveolar epithelial type 1; AT2: alveolar epithelial type 2; BMC: bone marrow-derived cells; CdM: conditioned media; EGF: epidermal growth factor; EPC: endothelial progenitor cell; HGF: hepatocyte growth factor; HSC: hematopoietic stem cell; hAEC: human amnion epithelial cell; hUC: human umbilical cord; IL: interleukin; i.n.: intranasal; i.p.: intraperitoneal; i.t.: intratracheal; i.v.: intravenous; KGF: keratinocyte growth factor; LVRS: lung volume reduction surgery; MMP-2: matrix metalloproteinase 2; MSC: mesenchymal stem cell; NO: nitric oxide; PGA: polyglycolic acid; TGF-*β*: transforming growth factor-*β*; TIMP: tissue inhibitor of metalloproteinase; VEGF: vascular endothelial growth factor.

## References

[1] Harding R, Maritz G (2012). Maternal and fetal origins of lung disease in adulthood. *Seminars in Fetal & Neonatal Medicine*.

[2] Wong PM, Lees AN, Louw J (2008). Emphysema in young adult survivors of moderate-to-severe bronchopulmonary dysplasia. *European Respiratory Journal*.

[3] Bourbon JR, Boucherat O, Boczkowski J, Crestani B, Delacourt C (2009). Bronchopulmonary dysplasia and emphysema: in search of common therapeutic targets. *Trends in Molecular Medicine*.

[4] Warburton D, Tefft D, Mailleux A (2001). Do lung remodeling, repair, and regeneration recapitulate respiratory ontogeny?. *American Journal of Respiratory and Critical Care Medicine*.

[5] Joss-Moore LA, Albertine KH, Lane RH (2011). Epigenetics and the developmental origins of lung disease. *Molecular Genetics and Metabolism*.

[6] Kinsella JP, Greenough A, Abman SH (2006). Bronchopulmonary dysplasia. *Lancet*.

[7] Schulzke SM, Pillow JJ (2010). The management of evolving bronchopulmonary dysplasia. *Paediatric Respiratory Reviews*.

[8] Henderson-Smart DJ, Hutchinson JL, Donoghue DA, Evans NJ, Simpson JM, Wright I (2006). Prenatal predictors of chronic lung disease in very preterm infants. *Archives of Disease in Childhood: Fetal and Neonatal Edition*.

[9] Ralser E, Mueller W, Haberland C (2012). Rehospitalization in the first 2 years of life in children born preterm. *Acta Paediatrica*.

[10] Friedrich L, Pitrez PMC, Stein RT, Goldani M, Tepper R, Jones MH (2007). Growth rate of lung function in healthy preterm infants. *American Journal of Respiratory and Critical Care Medicine*.

[11] Hjalmarson O, Sandberg K (2002). Abnormal lung function in healthy preterm infants. *American Journal of Respiratory and Critical Care Medicine*.

[12] Friedrich L, Stein RT, Pitrez PMC, Corso AL, Jones MH (2006). Reduced lung function in healthy preterm infants in the first months of life. *American Journal of Respiratory and Critical Care Medicine*.

[13] Gross SJ, Iannuzzi DM, Kveselis DA, Anbar RD (1998). Effect of preterm birth on pulmonary function at school age: A prospective controlled study. *Journal of Pediatrics*.

[14] Robin B, Kim YJ, Huth J (2004). Pulmonary Function in Bronchopulmonary Dysplasia. *Pediatric Pulmonology*.

[15] Tepper RS, Morgan WJ, Cota K, Taussig LM (1986). Expiratory flow limitation in infants with bronchopulmonary dysplasia. *Journal of Pediatrics*.

[16] Balinotti JE, Chakr VC, Tiller C (2010). Growth of lung parenchyma in infants and toddlers with chronic lung disease of infancy. *American Journal of Respiratory and Critical Care Medicine*.

[17] Narang I (2010). Review series: what goes around, comes around: childhood influences on later lung health? Long-term follow-up of infants with lung disease of prematurity. *Chronic Respiratory Disease*.

[18] Fawke J, Lum S, Kirkby J (2010). Lung function and respiratory symptoms at 11 years in children born extremely preterm: The EPICure study. *American Journal of Respiratory and Critical Care Medicine*.

[19] Doyle LW (2006). Respiratory function at age 8-9 years in extremely low birthweight/very preterm children born in Victoria in 1991-1992. *Pediatric Pulmonology*.

[20] Broström EB, Thunqvist P, Adenfelt G, Borling E, Katz-Salamon M (2010). Obstructive lung disease in children with mild to severe BPD. *Respiratory Medicine*.

[21] Smith LJ, Van Asperen PP, McKay KO, Selvadurai H, Fitzgerald DA (2008). Reduced exercise capacity in children born very preterm. *Pediatrics*.

[22] Welsh L, Kirkby J, Lum S (2010). The EPICure study: maximal exercise and physical activity in school children born extremely preterm. *Thorax*.

[23] Santuz P, Baraldi E, Zaramella P, Filippone M, Zacchello F (1995). Factors limiting exercise performance in long-term survivors of bronchopulmonary dysplasia. *American Journal of Respiratory and Critical Care Medicine*.

[24] Bolton CE, Stocks J, Hennessy E (2012). The EPICure Study: Association between hemodynamics and lung function at 11 years after extremely preterm birth. *Journal of Pediatrics*.

[25] Clemm H, Roksund O, Thorsen E, Eide GE, Markestad T, Halvorsen T (2012). Aerobic capacity and exercise performance in young people born extremely preterm. *Pediatrics*.

[26] Hacking DF, Gibson AM, Robertson C, Doyle LW Respiratory function at age 8-9 after extremely low birthweight or preterm birth in Victoria in 1997.

[27] Kaplan E, Yishay EB, Prais D (2012). Encouraging pulmonary outcome for surviving, neurologically intact extremely premature infants in the post surfactant era. *Chest*.

[28] Korhonen P, Laitinen J, Hyödynmaa E, Tammela O (2004). Respiratory outcome in school-aged, very-low-birth-weight children in the surfactant era. *Acta Paediatrica*.

[29] Kotecha SJ, Watkins WJ, Paranjothy S, Dunstan FD, Henderson AJ, Kotecha S (2012). Effect of late preterm birth on longitudinal lung spirometry in school age children and adolescents. *Thorax*.

[30] Kulasekaran K, Gray PH, Masters B (2007). Chronic lung disease of prematurity and respiratory outcome at eight years of age. *Journal of Paediatrics and Child Health*.

[31] Mai XM, Gäddlin PO, Nilsson L (2003). Asthma, lung function and allergy in 12-year-old children with very low birth weight: a prospective study. *Pediatric Allergy and Immunology*.

[32] Halvorsen T, Skadberg BT, Eide GE, Røksund OD, Carlsen KH, Bakke P (2004). Pulmonary outcome in adolescents of extreme preterm birth: a regional cohort study. *Acta Paediatrica, International Journal of Paediatrics*.

[33] Doyle LW, Faber B, Callanan C, Freezer N, Ford GW, Davis NM (2006). Bronchopulmonary dysplasia in very low birth weight subjects and lung function in late adolescence. *Pediatrics*.

[34] Narang I, Rosenthal M, Cremonesini D, Silverman M, Bush A (2008). Longitudinal evaluation of airway function 21 years after preterm birth. *American Journal of Respiratory and Critical Care Medicine*.

[35] Vrijlandt EJLE, Gerritsen J, Boezen HM, Grevink RG, Duiverman EJ (2006). Lung function and exercise capacity in young adults born prematurely. *American Journal of Respiratory and Critical Care Medicine*.

[36] Filippone M, Bonetto G, Corradi M, Frigo AC, Baraldi E (2012). Evidence of unexpected oxidative stress in airways of adolescents born very preterm. *European Respiratory Journal*.

[37] Aukland SM, Rosendahl K, Owens CM, Fosse KR, Eide GE, Halvorsen (2009). Neonatal bronchopulmonary dysplasia predicts abnormal pulmonary HRCT scans in long-term survivors of extreme preterm birth. *Thorax*.

[38] Sharma G, Goodwin J (2006). Effect of aging on respiratory system physiology and immunology.. *Clinical interventions in aging*.

[39] Wang L, Green FHY, Smiley-Jewell SM, Pinkerton KE (2010). Susceptibility of the aging lung to environmental injury. *Seminars in Respiratory and Critical Care Medicine*.

[40] Pinkerton KE, Green FHY, Harding R, Pinkerton KE, Plopper CG (2004). Normal Aging of the Lung. *The Lung: Development, Aging and the Environment*.

[41] Meyer KC (2005). Aging. *Proceedings of the American Thoracic Society*.

[42] Meyer KC, Ershler W, Rosenthal NS, Lu XG, Peterson K (1996). Immune dysregulation in the aging human lung. *American Journal of Respiratory and Critical Care Medicine*.

[43] Filippone M, Baraldi E (2011). On early life risk factors for COPD. *American Journal of Respiratory and Critical Care Medicine*.

[44] Jobe AH (2011). The new bronchopulmonary dysplasia. *Current Opinion in Pediatrics*.

[45] Agostini C (2010). Stem cell therapy for chronic lung diseases: hope and reality. *Respiratory Medicine*.

[46] Blaisdell CJ, Gail DB, Nabel EG (2009). National heart, lung, and blood institute perspective: lung progenitor and stem cells—gaps in knowledge and future opportunities. *Stem Cells*.

[47] Sueblinvong V, Weiss DJ (2010). Stem cells and cell therapy approaches in lung biology and diseases. *Translational Research*.

[48] Van Haaften T, Byrne R, Bonnet S (2009). Airway delivery of mesenchymal stem cells prevents arrested alveolar growth in neonatal lung injury in rats. *American Journal of Respiratory and Critical Care Medicine*.

[49] Baker CD, Ryan SL, Ingram DA, Seedorf GJ, Abman SH, Balasubramaniam V (2009). Endothelial colony-forming cells from preterm infants are increased and more susceptible to hyperoxia. *American Journal of Respiratory and Critical Care Medicine*.

[50] Borghesi A, Massa M, Campanelli R (2009). Circulating endothelial progenitor cells in preterm infants with bronchopulmonary dysplasia. *American Journal of Respiratory and Critical Care Medicine*.

[51] Liu X, Xie C (2012). Human endothelial progenitor cells isolated from COPD patients are dysfunctional. *Molecular and Cellular Biochemistry*.

[52] Timmermans F, Plum J, Yöder MC, Ingram DA, Vandekerckhove B, Case J (2009). Endothelial progenitor cells: identity defined?. *Journal of Cellular and Molecular Medicine*.

[53] Kim EK, Lee JH, Jeong HC (2012). Impaired colony-forming capacity of circulating endothelial progenitor cells in patients with emphysema. *The Tohoku Journal of Experimental Medicine*.

[54] Aslam M, Baveja R, Liang OD (2009). Bone marrow stromal cells attenuate lung injury in a murine model of neonatal chronic lung disease. *American Journal of Respiratory and Critical Care Medicine*.

[55] Tropea KA, Leder E, Aslam M (2012). Bronchioalveolar stem cells increase after mesenchymal stromal cell treatment in a mouse model of bronchopulmonary dysplasia. *American Journal of Physiology*.

[56] Zhang X, Wang H, Shi Y (2012). The role of bone marrow-derived mesenchymal stem cells in the prevention of hyperoxia-induced lung injury in newborn mice. *Cell Biology International*.

[57] Hansmann G, Fernandez-Gonzalez A, Aslam M (2012). Mesenchymal stem cell-mediated reversal of bronchopulmonary dysplasia and associated pulmonary hypertension. *Pulmonary Circulation*.

[58] Pierro M, Ionescu L, Montemurro T Short-term, long-term and paracrine effect of human umbilical cord-derived stem cells in lung injury prevention and repair in experimental bronchopulmonary dysplasia.

[59] Chang YS, Choi SJ, Sung DK (2011). Intratracheal transplantation of human umbilical cord blood derived mesenchymal stem cells dose-dependently attenuates hyperoxia-induced lung injury in neonatal rats. *Cell Transplantation*.

[60] Chang YS, Oh W, Choi SJ (2009). Human umbilical cord blood-derived mesenchymal stem cells attenuate hyperoxia-induced lung injury in neonatal rats. *Cell Transplantation*.

[61] Huh JW, Kim SY, Lee JH (2011). Bone marrow cells repair cigarette smoke-induced emphysema in rats. *American Journal of Physiology*.

[62] Zhen G, Liu H, Gu N, Zhang H, Xu Y, Zhang Z (2008). Mesenchymal stem cells transplantation protects against rat pulmonary emphysema. *Frontiers in Bioscience*.

[63] Shigemura N, Okumura M, Mizuno S (2006). Lung tissue engineering technique with adipose stromal cells improves surgical outcome for pulmonary emphysema. *American Journal of Respiratory and Critical Care Medicine*.

[64] Shigemura N, Okumura M, Mizuno S, Imanishi Y, Nakamura T, Sawa Y (2006). Autologous transplantation of adipose tissue-derived stromal cells ameliorates pulmonary emphysema. *American Journal of Transplantation*.

[65] Katsha AM, Ohkouchi S, Xin H (2011). Paracrine factors of multipotent stromal cells ameliorate lung injury in an elastase-induced emphysema model. *Molecular Therapy*.

[66] Kumamoto M, Nishiwaki T, Matsuo N, Kimura H, Matsushima K (2009). Minimally cultured bone marrow mesenchymal stem cells ameliorate fibrotic lung injury. *European Respiratory Journal*.

[67] Moodley Y, Atienza D, Manuelpillai U (2009). Human umbilical cord mesenchymal stem cells reduce fibrosis of bleomycin-induced lung injury. *American Journal of Pathology*.

[68] Ortiz LA, DuTreil M, Fattman C (2007). Interleukin 1 receptor antagonist mediates the antiinflammatory and antifibrotic effect of mesenchymal stem cells during lung injury. *Proceedings of the National Academy of Sciences of the United States of America*.

[69] Ortiz LA, Gambelli F, McBride C (2003). Mesenchymal stem cell engraftment in lung is enhanced in response to bleomycin exposure and ameliorates its fibrotic effects. *Proceedings of the National Academy of Sciences of the United States of America*.

[70] Rojas M, Xu J, Woods CR (2005). Bone marrow-derived mesenchymal stem cells in repair of the injured lung. *American Journal of Respiratory Cell and Molecular Biology*.

[71] Lee JW, Fang X, Krasnodembskaya A, Howard JP, Matthay MA (2011). Concise review: mesenchymal stem cells for acute lung injury: role of paracrine soluble factors. *Stem Cells*.

[72] Balasubramaniam V, Ryan SL, Seedorf GJ (2010). Bone marrow-derived angiogenic cells restore lung alveolar and vascular structure after neonatal hyperoxia in infant mice. *American Journal of Physiology*.

[73] Vosdoganes P, Hodges RJ, Lim R (2011). Human amnion epithelial cells as a treatment for inflammation-induced fetal lung injury in sheep. *American Journal of Obstetrics and Gynecology*.

[74] Moodley Y, Ilancheran S, Samuel C (2010). Human amnion epithelial cell transplantation abrogates lung fibrosis and augments repair. *American Journal of Respiratory and Critical Care Medicine*.

[75] Murphy S, Lim R, Dickinson H (2011). Human amnion epithelial cells prevent bleomycin-induced lung injury and preserve lung function. *Cell Transplantation*.

[76] Hegab AE, Kubo H, Fujino N (2010). Isolation and characterization of murine multipotent lung stem cells. *Stem Cells and Development*.

[77] Wang D, Morales JE, Calame DG, Alcorn JL, Wetsel RA (2010). Transplantation of human embryonic stem cell-derived alveolar epithelial type II cells abrogates acute lung injury in mice. *Molecular Therapy*.

[78] Aguilar S, Scotton CJ, McNulty K (2009). Bone marrow stem cells expressing keratinocyte growth factor via an inducible lentivirus protects against bleomycin-induced pulmonary fibrosis. *PLoS ONE*.

